# Sustained clinical remission of epidermolysis bullosa simplex due to a pathogenic KRT14 variant in a 7-year-old girl

**DOI:** 10.1016/j.jdcr.2026.05.039

**Published:** 2026-05-22

**Authors:** Simona Abou Tayeh, Nakhle Ayoub

**Affiliations:** a School of Medicine and Medical Sciences, Holy Spirit University of Kaslik, Jounieh, Lebanon; b Dermatology Department, Hôtel-Dieu de France, Beirut, Lebanon

**Keywords:** Epidermolysis Bullosa Simplex, isotretinoin, keratin mutations, retinoids, skin fragility disorders

## Introduction

Epidermolysis Bullosa Simplex (EBS) is a rare genetic condition characterized by skin fragility and intraepidermal blistering, typically induced by minor mechanical trauma.^1^ EBS is the most common form^2^ and distinguished from other forms by the cleavage plane, which is within basal keratinocytes of the epidermis rather than at the dermal-epidermal junction or below. The severity of blistering ranges from limited to hands and feet to widespread involvement. Additional features can include hyperkeratosis of the palms and soles (keratoderma), nail dystrophy, milia, and hyper- and/or hypopigmentation.^1^

EBS is primarily caused by dominant mutations in the KRT5 or KRT14 genes, which encode keratin 5 and keratin 14, respectively. These keratins are essential for the structural integrity of basal keratinocytes. Mutations can impact the structure of these proteins, causing keratin aggregates in keratinocytes, triggering endoplasmic reticulum stress and leading to the release of pro-inflammatory molecules.^3^ Intraepidermal ruptures occur and lead to loss of tissue integrity and severe blistering of the epidermis upon mechanical trauma or traction.^4^ Molecular studies have demonstrated upregulation of pro-inflammatory cytokines, leading to activation of downstream signaling cascades. These promote keratinocyte stress responses and impair cell–cell adhesion, thereby exacerbating epidermal fragility.

Diagnosis is based on clinical presentation, although genetic testing for KRT5 and KRT14 mutations is definitive and recommended for accurate subtype classification.^1^

There is currently no curative therapy for EBS; management is symptomatic^2^ including: prevention of trauma and friction, wound care with non-adhesive dressings and topical agents to promote healing and prevent infection, management of pain and pruritus, treating secondary infections. Given the absence of effective disease-modifying therapies, alternative targeted approaches warrant further clinical exploration.

We report the case of a patient with clinically and genetically confirmed epidermolysis bullosa simplex who demonstrated a notable clinical response following treatment with a novel therapeutic approach.

## Case report

A 7-year-old girl (weight: 22 kg) presented with a history of recurrent cutaneous blistering that began in infancy. The first blister appeared at the age of 9 months, shortly after she started bearing weight on her feet. She was initially evaluated in the emergency department, where the lesion was drained and interpreted as a burn injury.

Following this episode, blistering progressively increased in frequency. Lesions occurred predominantly on the plantar surfaces of the feet and in the diaper area. Blisters developed after minimal mechanical trauma and were exacerbated by heat and sweating. Management consisted of repetitive drainage of bullae and general wound care to prevent secondary infection. Throughout early childhood, the disease caused significant functional impairment. The patient had trouble walking, particularly during warmer seasons, when blister formation was frequent and severe. These limitations significantly affected her quality of life. The patient was born to non-consanguineous parents, with no family history of similar skin conditions.

*Genetic testing* was performed using whole exome sequencing on a peripheral blood sample. Analysis identified a heterozygous pathogenic variant in the *KRT14* gene (NM_000526.4:c.1151T>C; p.Leu384Pro), consistent with autosomal dominant epidermolysis bullosa simplex. Importantly, no pathogenic or likely pathogenic variants were identified in other genes associated with epidermolysis bullosa, including COL7A1. (Detailed genetic findings are provided in Supplementary File 1, available via Mendeley at https://data.mendeley.com/datasets/brdjzm74pw/1.)

This variant has been previously reported in affected individuals and confirmed the diagnosis of generalized intermediate epidermolysis bullosa simplex, an autosomal dominant disorder caused by heterozygous mutations in *KRT14*.

The family was informed that spontaneous improvement often occurs at puberty. Expectant management was recommended. At the age of 7 years, following referral by her pediatrician due to increasing in severity of her presentation, the patient was reevaluated. For the first time since disease onset, a medical treatment was initiated. This regimen included oral isotretinoin at a dose of 1 pill of 5 mg every 5 days, which is being continued on a long-term basis. Since initiation of therapy, there has been a marked and progressive reduction in blister frequency. We report clinical remission for 120 consecutive days. At the time of revision, extended follow-up totaling approximately 225 days revealed sustained clinical remission, with no new blister formation reported by the patient’s caregiver. The temporal association between treatment initiation and the marked reduction in blister frequency suggests a therapeutic benefit beyond natural disease progression.

## Discussion

The diagnosis in our case is supported by molecular confirmation of a pathogenic variant in KRT14, with no evidence of variants in COL7A1. This effectively excludes dystrophic forms of EB and argues against a combined keratin-collagen 7 genotype. This case highlights the potential role of a targeted therapeutic strategy in epidermolysis bullosa simplex.

Topical therapies previously used include 1% diacerein cream, topical sirolimus, and topical cannabidiol oil.^1^ Systemic therapies include oral tetracyclines^5^ and cyproheptadine which has been used for its anti-pruritic effect.^1^ Botulinum toxin injections into the feet have been reported in case series to reduce blistering and pain.^1^ It is important to note that none of these agents are approved for EBS.

Retinoids modulate transcription of keratin genes through nuclear retinoic acid receptors. Experimental studies demonstrate that retinoic acid can alter keratin expression profiles, downregulating hyperproliferative or stress-associated keratins while promoting a more normalized differentiation pattern. This regulatory effect may reduce the burden of mutant keratin accumulation in basal keratinocytes.^6^ Vitamin A promotes controlled keratinocyte differentiation and epidermal turnover. Enhanced renewal of basal keratinocytes may facilitate replacement of structurally fragile cells, potentially reducing blister frequency and severity in milder EBS phenotypes. From a mechanistic perspective, the *KRT14* p.Leu384Pro variant is expected to disrupt the assembly and stability of keratin intermediate filaments within basal keratinocytes, leading to increased cellular fragility under mechanical stress.^7^ *In vitro* studies^8^ have shown that retinoids can influence intermediate filament organization, decreasing cytoplasmic keratin aggregation. This effect may contribute to improved cytoskeletal resilience in keratinocytes harboring pathogenic mutations. It is therefore plausible that retinoid-mediated changes in keratin expression or filament organization could reduce cytoskeletal stress or keratin aggregation in cells harboring mutant *KRT14*, thereby decreasing susceptibility to blister formation.

A case report^9^ described the use of a topical retinoid (tazarotene) and oral retinoid (Acitretin) with a clinical and symptomatic response in a pediatric patient. From a pharmacologic perspective, isotretinoin has a shorter elimination half-life compared with acitretin, which may allow greater flexibility in dosing and easier discontinuation, particularly in pediatric patients. Notably, the regimen used in this case was substantially lower than doses typically reported, raising the possibility that even minimal retinoid exposure may exert regulatory effects on keratinocyte differentiation or inflammatory pathways. Effectively, retinoids exert transcriptional regulatory effects that may occur at relatively low exposure levels, and intermittent dosing may allow cumulative biologic effects on epidermal differentiation and inflammatory signaling pathways.^6^ This raises the possibility that even minimal retinoid exposure could influence disease expression.

Isotretinoin exerts anti-inflammatory effects by down-regulating Toll-like receptor-2 expression on monocytes and decreasing pro-inflammatory cytokines such as IL-8 and TNF-α, as well as inhibiting leukocyte migration and modulating transcriptional pathways like. These immune-modulating actions overlap with inflammatory pathways implicated in the pathogenesis of Epidermolysis Bullosa Simplex (MAPK, TNF-α, and IL-1 signaling), suggesting a theoretical effect of isotretinoin on inflammation in EBS.^10^ Moreover, Vitamin A and systemic retinoids require cautious use due to dose-dependent toxicity, teratogenicity, and potential worsening of skin fragility.

The variable effects of retinoids across EB subtypes highlight the importance of precise genetic diagnosis. In collagen 7-deficient dystrophic EB for example, retinoids may worsen skin fragility through nonspecific epidermal disruption.^11^ By contrast, in KRT14-variant EBS, where the mutant protein is under direct retinoid transcriptional control, the therapeutic rationale is fundamentally different. Our case illustrates that retinoid responsiveness in EB is not a class property of the disease but rather a molecularly specific phenomenon.

This case showed that even in genetically confirmed epidermolysis bullosa simplex, significant improvement in blistering can be achieved with targeted interventions. This report describes a single patient, which limits generalizability. Although electron microscopy and immunofluorescence mapping were not performed, the diagnosis is supported by the clinical presentation and confirmatory genetic findings. Notably, the sustained remission observed over an extended follow-up period of approximately 225 days is encouraging, however, improvement could be influenced by the natural tendency of intermediate EBS to become less severe with age, making it difficult to attribute the response solely to treatment so a causal relationship with isotretinoin cannot be definitively established. In addition, long-term outcomes, including durability of clinical remission and potential adverse effects of prolonged intermittent isotretinoin therapy should be investigated further.

## Conclusion

In conclusion, children with epidermolysis bullosa simplex, often referred to as “butterfly children” due to their fragile skin, are constantly suffering from functional limitations, affecting their childhood. This case where intermittent oral isotretinoin was associated with a remarkable reduction in blistering, highlights the importance of individualized treatment strategies that can restore not only skin integrity but also hope for these vulnerable children, highlighting the need for further research to evaluate long-term safety and efficacy.

## Conflicts of interest

None disclosed.

## Supplementary data

Genetic testing
