# Supplementary material for: Sustained clinical remission of epidermolysis bullosa simplex due to a pathogenic KRT14 variant in a 7-year-old girl
Source: JAAD Case Rep. 2026 May 22;74:95–7. doi: 10.1016/j.jdcr.2026.05.039 (PMC13316663; doi:10.1016/j.jdcr.2026.05.039)
Supplement: Genetic testing [file mmc1.pdf]

Department of Human Genetics

Molecular Genetics Report

Sample Type: Blood
Sample Received on: 02 Sept. 2023
Report Date: 28 Sept. 2023

Patient File Number: [redacted]
First Name: [redacted]
Last Name: [redacted]
D.O.B: [redacted]
Referred by: [redacted]

Methodology:

We performed Whole Exome sequencing (WES) in the patient [redacted]. Sample was prepared and enriched to the manufacturer's standard protocol. Concentration of each library was determined using Agilent's QPCR NGS Library Quantification Kit (G4880A). Samples were pooled prior to sequencing with each sample at a final concentration of 10nM. Sequencing was performed on the Illumina HiSeq2000 platform using TruSeq v3 chemistry.

Mapping and Alignment: Read files (Fastq) were generated from the sequencing platform via the manufacturer's proprietary software. Reads were mapped to their location in the most recent build of the human genome (hg19/b37) using the Burrows-Wheeler Aligner (BWA) package, version 0.6.1. Local realignment of the mapped reads around potential insertion/deletion (indel) sites was carried out with the Genome Analysis Tool Kit (GATK) version 1.6. This algorithm ensures that the alignment has the minimum number of mismatching bases across the reads. Thus reducing false-positive SNP calls around indels, and also accurately determining indel length. Duplicate reads were marked using Picard version 1.62. This removes reads likely to be the result of PCR bias. Such PCR artefacts can introduce false positive SNP calls. Reads are not removed from the alignment but are not considered further in the analysis. Additional BAM file manipulations were performed with Samtools 0.1.18. Base quality (Phred scale) scores were recalibrated using GATK's covariance recalibration. This improves the accuracy of the base quality metrics that in turn improves the quality of variant calls. SNP and indel variants were called using the GATK Unified Genotyper for each sample (4). SNP novelty is determined against dbSNP.

Variant Analysis: Variants were annotated with gene and gene function data from Ensembl. This shows which genes and transcripts are affected by variations, and whether these variants are likely to cause significant functional problems. Known variants from dbSNP (Release 135) are annotated within the dataset so that novel variants with serious predicted consequences may be rapidly identified.

The prioritization and interpretation of genetic variants have been performed by qualified personnel considering clinical, genetic scientific and population criteria, contrasting this data with a series of population databases, diseases and genes (OMIM, ExAC, 1000G, gnomAD, HGMD, ClinVar, ClinGen, etc), and using in silico pathogenicity prediction programs (SIFT, Polyphen, Mutation Taster, etc). Variants in coding and splicing regions up to ± 10 bp from exon, and those non-coding ones previously classified as pathogenic or probably pathogenic, have been analyzed.

The classification of genetic variants based on its clinical implication has been carried out according to the recommendations of the American College of Medical Genetics (ACMG) (Richards et al., 2015). The categorization of genetic variants established by the ACMG are the following: pathogenic (P), likely pathogenic (LP), variant of uncertain significance (VUS), probably benign and benign (B).

The report includes those genetic variants directly related to the clinical indication of the study. "Relevant Variants related to the clinical indication" are those genetic variants classified as pathogenic or probably pathogenic identified in genes directly related with the clinical indication, and those ones that could be related to the clinical indication due to their biological characteristics, but without clear clinical evidence.

Results:

The Whole Exome Sequencing analysis for the patient [redacted] was performed. Genes involved in skin disorders were deeply analyzed (Annex 1). WES data was analyzed to assess the presence of pathogenic variants involved in autosomal recessive diseases; autosomal dominant; and X-linked diseases.

The patient [redacted] was found heterozygous for a variant in the *KRT14* gene. According to the algorithms developed to predict the effect of a variant on protein structure and function and according to the ACMG recommendations, this variant was classified as of Pathogenic. This variant was already reported in affected patients.

Gene	cHGVS	pHGVS	Zygosity	Classification
KRT14	NM_000526.4:c.1151T>C	p.Leu384Pro	heterozygous	Pathogenic Known

- Generalized intermediate epidermolysis bullosa simplex-1B (EBS1B) is caused by heterozygous mutation in the *KRT14* gene. It is an autosomal dominant disorder of skin in which intraepidermal blistering occurs after minor mechanical trauma. Skin blistering is generalized, begins at birth, and is worsened by heat, humidity, and sweating. The tendency to blistering diminishes in adolescence, when it may become localized to hands and feet. Intermediate EBS has previously been known as the Koebner type.

Additional (carrier) Findings

Carrier status determines the proband's risk for passing inherited genetic condition(s) to the children. Carriers are typically healthy/ asymptomatic. When an individual is found to be a carrier of a genetic condition, his or her relatives are at risk of carrying the same variant or another one in the same gene.

The patient [redacted] is heterozygous carrier for the following pathogenic / Likely_Pathogenic variants in genes involved in autosomal recessive diseases:

Gene	cHGVS	pHGVS	Classification	Disease
ALG12	NM_024105.3:c.664+1G>A	------	pathogenic	Congenital disorder of glycosylation type Ig
ATG7	NM_006395.2:c.1277C>T	p.Pro426Leu	Pathogenic	Spinocerebellar ataxia type 31
DLX5	NM_005221.5:c.518C>T	p.Ser173Leu	Likely pathogenic	Split-hand/foot malformation + deafness

RECOMMENDATIONS:

- Genetic counseling is warranted to review the implications of these results.
- Genotype-Phenotype correlation is recommended.

Disclaimers:

The performed test aims to assess the variants detected in the genes listed in OMIM as involved in Human Diseases. This test does not rule out the presence of pathogenic variants not previously reported.

WES does not enable the detection of specific genetic variants such as large deletions/duplications, This assay achieves more than 95% analytical sensitivity and specificity for single nucleotide variants, insertions and deletions smaller than 15bp in length, and deletions and duplications affecting several exons. Coverage data obtained from this assay enables to assess copy numbers at the resolution of few exons. Single-exon copy number events may not be analyzed due to inherent sequence properties or isolated reduction in data quality. Certain types of variants, such as structural rearrangements (e.g. inversions, gene conversion events, translocations, etc.) or variants embedded in sequence with complex architecture (e.g. short tandem repeats or segmental duplications), may not be detected. Poor coverage of some genetic region may also lead to false negative results. Poor coverage of some genetic region may also lead to false negative results.

The interpretation of the exome is based on our current understanding of specific genes. This interpretation may change over time as more information about the genes or the addition of other family members become available.

Pr. André MEGARBANE
Medical Genetics
M/668

Andre MEGARBANE, MD, PhD
Professor & Chairman

ANNEX 1

ANNEX 1 :

Skin Disease Panel:

AAGAB, ABCA12, ABCB6, ABCC9, ABHD5, ACD, ACTA2, ACVRL1, ADA2, ADAM10, ADAMTS3, ADAR, AGPAT2, ALDH18A1, ALOX12B, ALOXE3, ANAPC1, ANGPT1, ANTXR1, AP3B1, AP3D1, APCDD1, AQP5, ATP2A2, ATP2C1, ATP6V0A2, ATP6V1A, ATP6V1E1, AXIN2, BANF1, BCS1L, BLM, BLOC1S3, BLOC1S5, BLOC1S6, BRAF, BSCL2, CARD14, CAST, CAV1, CAVIN1, CCBE1, CCM2, CDH3, CDSN, CELSR1, CERS3, CLDN1, COL17A1, COL3A1, COL7A1, CSTA, CTC1, CTSC, CYP4F22, DCT, DDB2, DKC1, DSG1, DSG4, DSP, DST, DTNBP1, EDA, EDAR, EDARADD, EDN3, EDNRB, EFEMP2, ELN, ENG, ENPP1, EPG5, EPHB4, ERCC1, ERCC2, ERCC3, ERCC4, ERCC5, ERCC6, ERCC8, EXPH5, F12, FAT4, FBLN5, FBN1, FDPS, FERMT1, FGF10, FGFR2, FGFR3, FLG, FLG2, FLT4, FOXC2, GATA2, GDF2, GJA1, GJB2, GJB3, GJB4, GJB6, GJC2, GORAB, GPR143, GRHL2, GTF2E2, GTF2H5, GUCY1A1, HOXC13, HPS1, HPS3, HPS4, HPS5, HPS6, HR, ITGA3, ITGA6, ITGB4, JUP, KCNJ6, KDF1, KDSR, KIF11, KIT, KITLG, KLHL24, KNG1, KREMEN1, KRIT1, KRT1, KRT10, KRT14, KRT16, KRT17, KRT2, KRT25, KRT5, KRT6A, KRT6B, KRT6C, KRT74, KRT81, KRT83, KRT85, KRT86, KRT9, LAMA3, LAMB3, LAMC2, LIG4, LIPH, LMNA, LORICRIN, LPAR6, LRMDA, LRP6, LSS, LTBP4, LYST, LZTR1, MAP2K1, MBTPS2, MC1R, MITF, MLPH, MPLKIP, MSX1, MVD, MVK, MYO5A, NECTIN1, NECTIN4, NF1, NF2, NFKB2, NFKBIA, NHP2, NIPAL4, NOP10, NPM1, OCA2, OFD1, PARN, PAX3, PAX9, PDCD10, PDGFRB, PERP, PIEZO1, PIK3R1, PKP1, PLEC, PLG, PLIN1, PMVK, PNPLA1, POFUT1, POGLUT1, POLD1, POLH, POLR3A, PORCN, PPARG, PRKAR1A, PSENEN, PSMB8, PTEN, PTPN11, PYCR1, RAB27A, RAF1, RASA1, RECQL4, RHBDF2, RMRP, RNF213, RTEL1, SASH1, SDR9C7, SERPINB7, SERPING1, SLC24A5, SLC25A24, SLC27A4, SLC2A10, SLC45A2, SLC6A19, SLURP1, SMAD4, SMARCB1, SOX10, SOX18, SPINK5, SPRED1, ST14, ST3GAL5, STK11, STS, SULT2B1, TEK, TERC, TERT, TGM1, TGM5, THSD1, TINF2, TOP3A, TP63, TRPS1, TRPV3, TSPEAR, TYR, TYRP1, USB1, UVSSA, VEGFC, WNT10A, WNT10B, WRAP53, WRN, XPA, XPC, YY1AP1, ZMPSTE24
